# SSTR-directed peptide receptor radionuclide therapy for recurrent meningiomas: analysis of safety, efficacy and prognostic factors

**DOI:** 10.1007/s00259-025-07336-6

**Published:** 2025-06-02

**Authors:** Natalie Hasenauer, Miriam Müller, Heribert Hänscheid, Sebastian E. Serfling, Kerstin Michalski, Marieke Heinrich, Bülent Polat, Andreas K. Buck, Rudolf A. Werner, Philipp E. Hartrampf

**Affiliations:** 1 Department of Nuclear Medicine, University Hospital Würzburg, Oberdürrbacherstraße 6, 97080 Würzburg, Germany; 2 Department of Radiation Oncology, University Hospital Wuerzburg, Würzburg, Germany; 3 Department of Nuclear Medicine, University Hospital, Ludwig-Maximilians-Universitaet Muenchen, Munich, Germany

**Keywords:** Meningioma, PRRT, Lu177, DOTATOC, SSTR, Peptide receptor radionuclide therapy

## Abstract

**Purpose:**

This study evaluates safety and efficacy of peptide receptor radionuclide therapy (PRRT) as a stand-alone treatment for recurrent meningiomas and investigates the prognostic value of laboratory markers and quantitative PET parameters.

**Methods:**

The single-center study includes 32 patients with recurrent meningioma, who underwent PRRT with [^177^Lu]Lu-DOTATOC/-TATE. Pre-treatment assessments comprised [^68^ Ga]Ga-DOTATOC PET imaging, routine hematology and serum chemistry analysis. Outcomes including progression-free survival (PFS), overall survival (OS), and treatment related toxicity, were retrospectively evaluated using Kaplan–Meier survival analysis and Cox regression models.

**Results:**

PRRT showed only mild hematological and renal toxicity, with most adverse events being low-grade (87%). OS was significantly shorter in patients with WHO grade III meningiomas (10 months) compared to grade I (not reached, HR 4.77, *p* < 0.01) and grade II (47 months, HR 4.05, *p* = 0.01). Similarly, PFS was shorter in patients with WHO grade III meningiomas (4.5 months) compared to grade I (17 months, HR 6.47, *p* < 0.001) and grade II (17 months, HR 2.71, *p* = 0.02). In multivariable analysis, only higher WHO grade was an independent predictor of disease progression, while baseline PET and laboratory parameters showed no consistent association. Furthermore, increase of SSTR-positive tumor volume in follow-up PET was associated with shorter PFS (HR 1.02, *p* = 0.02).

**Conclusion:**

PRRT is a safe treatment option and appears to have a favourable effect in patients with recurrent meningiomas. WHO tumor grade is the strongest predictor of PFS and OS, while baseline PET parameters appear to have no prognostic value.

**Supplementary Information:**

The online version contains supplementary material available at 10.1007/s00259-025-07336-6.

## Introduction

Meningiomas are the most common primary intracranial tumor in adults, accounting for approximately 30% of cases [1]. Meningiomas are classified into three grades (WHO 1–3) based on mitotic rate, brain invasion or histological features [2]. Low-grade meningiomas are often characterized by slow growth and a favorable prognosis. However, depending on their grading, location and size, meningiomas can cause serious morbidity and mortality. Surgical resection or external beam radiotherapy are the primary treatment options for progressive or symptomatic meningiomas [3]. However, when surgical resection or external radiotherapy are no longer applicable, recurrent and refractory meningiomas continue to pose an unresolved therapeutic challenge. In recent years, a number of pharmacological treatment options has been investigated, with anti-angiogenic drugs showing some effects regarding growth inhibition [4]. However, systemic treatment options and reliable data are still limited and there is no established standard of care to effectively control disease progression in these cases. Peptide receptor radionuclide therapy (PRRT) has been used for several years in oncological nuclear medicine for the treatment of metastatic neuroendocrine tumors [5]. PRRT targets somatostatin receptors (SSTRs), which are overexpressed on the surface of various tumor cells. Radiolabeled somatostatin analogues, such as DOTA(0)-Phe(1)-Tyr(3)octreotide (DOTATOC), bind to SSTRs, thus enabling the targeted internal irradiation of tumor tissue via β-emitting radionuclides like [^1^⁷⁷Lu]. The short tissue penetration range of β- radiation allows for high-dose irradiation of tumor tissue while largely sparing surrounding healthy structures [6]. Similar to neuroendocrine neoplasms, meningiomas frequently show high levels of SSTR expression [7–9], which makes them susceptible to targeted treatment with radiolabeled somatostatin analogues.

Previous studies have reported on efficacy and toxicity of PRRT as a stand-alone treatment in recurrent meningiomas [10–18]. However, most studies included small or heterogenous patient cohorts, lacked standardized treatment approaches, and did not asses the prognostic value of baseline PET imaging or laboratory parameters. The objective of the present study is to confirm the potential of PRRT as a stand-alone treatment option for patients diagnosed with recurrent or refractory meningioma in our cohort. In addition to analyzing safety and efficacy of the treatment, this study focuses on the evaluation of the prognostic value of laboratory chemical markers and quantitative parameters from baseline and follow-up PET scans. Moreover, this study investigates the association between tumor dose and treatment response.

## Material and methods

### Patient cohort

**Fig. 1:**
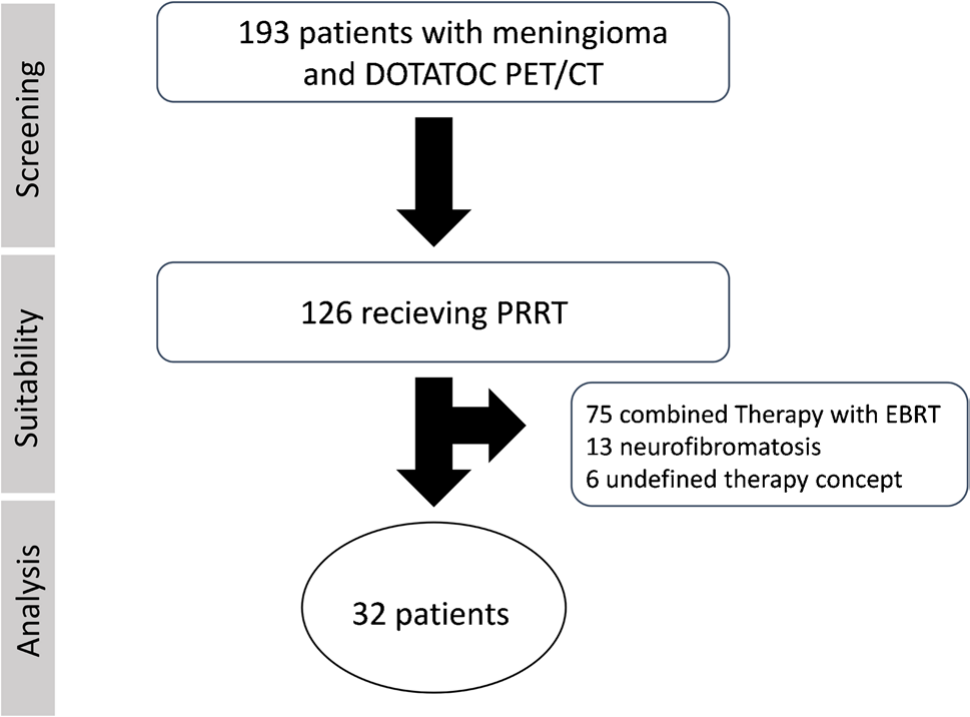
Flowchart of screening and patient selection. PET/CT positron emission tomography/computed tomography, PRRT peptide receptor radionuclide therapy,. EBRT external beam radiation therapy

**Fig. 2:**
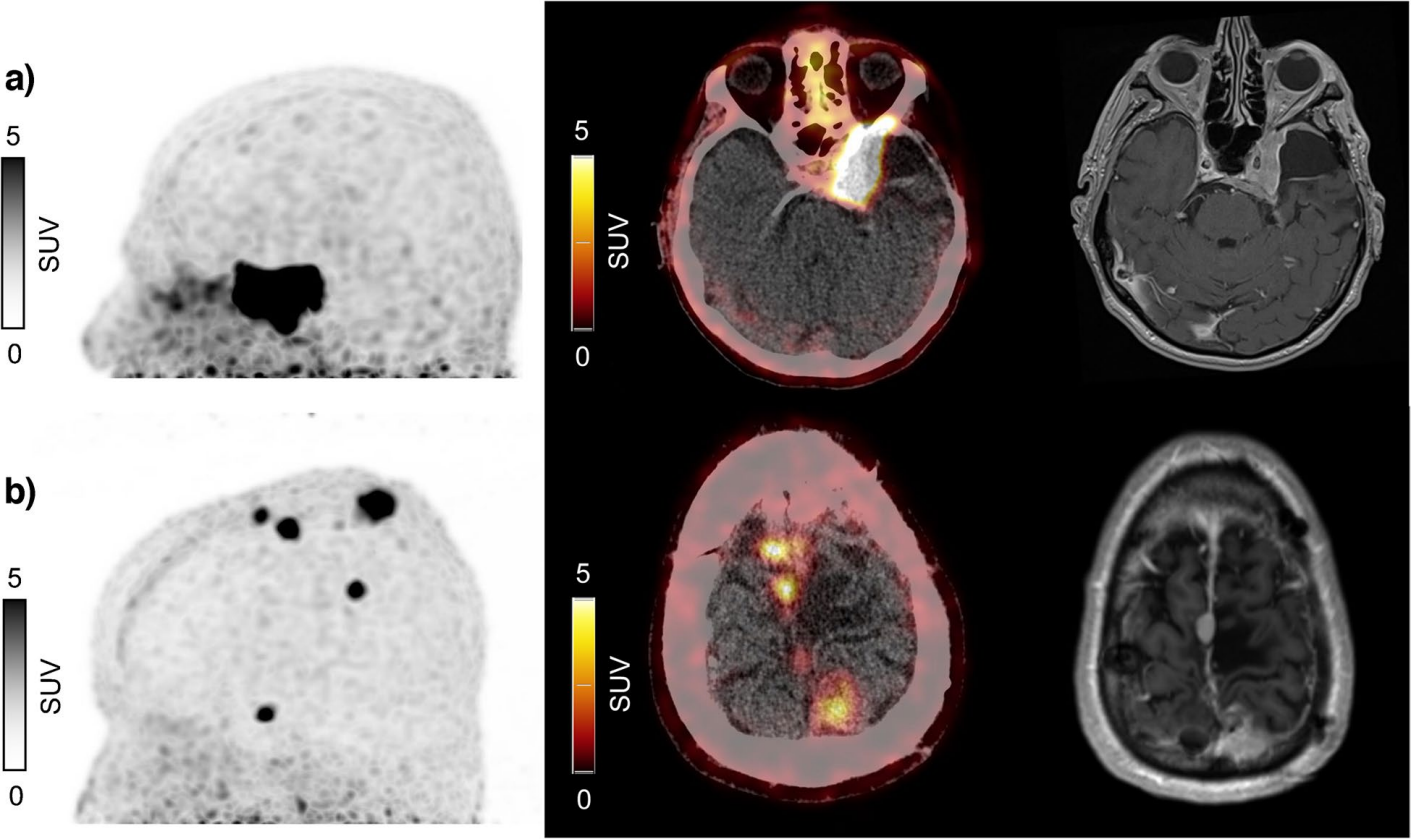
Left: Maximum intensity projection (MIP) representation of DOTATOC PET scan in lateral view. Middle: transversal slice of PET Scan after fusion with low-dose CT scan. Right: Transversal slice of pretherapeutic MRI (T1 post KM) (**a**) Example of a patient with a single lesion and (**b**) a patient with multiple lesions

In this single-center study, we screened 193 subjects who underwent [^68^ Ga]Ga-DOTATOC positron emission tomography (PET) at our center between 2013 and 2022. Of these, 126 patients were identified who underwent PRRT for recurrent meningioma. Patients were eligible for inclusion if they had histologically or radiologically confirmed recurrent or progressive meningioma, and received at least one cycle of PRRT at our institution. Patients were excluded if they received PRRT in combination with other systemic therapies or external beam radiation. This was done to ensure a homogeneous treatment protocol and to allow for a focused evaluation of PRRT as a stand-alone treatment. Furthermore, patients with neurofibromatosis were excluded, as neurofibromatosis-associated meningiomas may differ from sporadic cases in terms of underlying tumor biology and may therefore follow a different clinical course. A total of 32 patients were finally included in the analysis (Fig. 1). Prior to therapy, all participants provided consent for data analysis. Due to the retrospective character of the study, the Ethics Committee waived the need of consent (waiver number 2022072501). The median age at first cycle of therapy was 64.5 years (30—84). 59% of subjects were female. 31% (10/32) of the patients had a WHO grade I meningioma, 44% (14/32) grade II and 19% (6/32) grade III tumors. In two cases (6%), no histopathological report was available. Most patients had received multiple treatments prior to PRRT, with surgical resection in 94% and external beam radiotherapy in 72% of the patients (Table 1). Different morphological growth patterns were observed, 25% had one single lesion and 75% more than one lesion (Fig. 2). In one case, systematic metastases were found (lung, liver bone).

**Table 1:** Patient characteristics and baseline parameter

Clinical Variable	
Age at first cycle of PRRT (years) [median (range)]	64.5 (30—84)
Female [*n* (%)]	19 (59)
WHO grading at time of therapy	*n* (%)
WHO 1	10 (31)
WHO 2	14 (44)
WHO 3	6 (19)
Unknown WHO grading	2 (6)
Growth pattern	*n* (%)
single lesion	8 (25)
several larger lesions	11 (34)
Broadly diffuse lesion	3 (9)
Multiple small lesions	9 (28)
metastatic	1 (3)
Prior therapies	
Surgical resection [*n* (%)]	30 (94)
Number of resections [median (range)]	2 (1—6)
External beam radiation therapy [*n* (%)]	23 (72)
Number of prior EBRT [median (range)]	1 (1—4)
Treatment regiment	median (range)
Cumulative activity (GBq)	22.4 (6.9—60.3)
Treatment cycles/patient	2.5 (1—8)
Treatment cycles per patient	*n* (%)
One cycle	7 (22)
Two cycles	9 (28)
Three cycles	4 (13)
Four cycles	10 (31)
Six cycles	1 (3)
Eight cycles	1 (3)
Baseline laboratory values	median (range)
AST (U/l)	20 (13—42)
Alk. Phosphatase (U/l)	69 (38—172)
CRP (mg/dl)	0.22 (< 0.1—7.62)
Bilirubin (mg/dl)	0.35 (< 0.2–0.8)
LDH (U/l)	191 (158—427)
Creatinine (mg/dl)	0.77 (0.36—1.29)
eGFR (CKD-EPI) (ml/min/1,73 cm^2^)	92 (43—123)
Hemoglobin (g/dl)	13.65 (8.8—16.8)
Leucocytes (n*1000/µl)	7.2 (2.3—11.7)
Thrombocytes (n*1000/µl)	253 (105—495)
Baseline PET parameters	median (range)
SSTR-TV (cm^3^)	25 (5—363)
TL-SSTR (cm^3^)	159 (9—1884)
SUV_max_	25 (5—297)
SUV_peak_	13 (3—153)
SUV_mean_	4 (1—57)

PRRT peptide receptor radionuclide therapy, CRP C-reactive protein, AST aspartate aminotransferase, AP alkaline phosphatase, LDH lactate dehydrogenase, eGFR estimated glomerular filtration rate, SSTR-TV somatostatin receptor positive tumor volume, TL-SSTR total lesion SSTR expression, SUV standardized uptake value, EBRT external beam radiation therapy

### Pre-therapy work-up

Prior to PRRT all patients received [^68^ Ga]Ga-DOTATOC PET scan of the brain with low-dose computed tomography (CT) for attenuation correction and anatomical co-registration (see supplement material). The median injected activity was 112 MBq (60—231 MBq). The median time between [^68^ Ga]Ga-DOTATOC PET and initiation of PRRT was 41 days. Blood samples were taken on the day of admission, before initiation of therapy, including routine hematology (leucocytes, hemoglobin, and platelets) and serum chemistry for analysis of serum creatinine, estimated glomerular filtration rate (eGFR), C-reactive protein (CRP), alkaline phosphatase (AP), aspartate aminotransferase (AST), bilirubin, and lactate dehydrogenase (LDH). Hematology was examined using an automated analyzer (Sysmex XN-9000, Kobe, Japan) and serum chemistry was analyzed using a fully automated modular analyzer (Roche Cobas, Basel, Switzerland). Patient histories were retrospectively reviewed from medical records.

### PET image analysis

The freely available Beth Israel plugin for FIJI (Fiji Is Just ImageJ) software was used for image analysis [19, 20]. Focal tracer accumulation above background and outside tissue with physiological uptake, such as pituitary gland, was considered positive for recurrent meningioma. In addition to the maximum (SUV_max_) and peak (SUV_peak_) standardized uptake values, total SSTR-positive tumor volume (SSTR-TV) was estimated using a semi-automatic analysis with fixed SUV threshold of 3 and the mean SUV value (SUV_mean_) in SSTR-TV was determined. An expert SSTR PET reader (P.E.H.) reviewed all lesions. The SSTR-TV was multiplied by the SUV_mean_ to calculate the total lesion SSTR (TL-SSTR). When a follow-up PET scan was available (13 cases), the percentage changes between baseline and follow-up (Δ) were calculated for all quantitative PET parameters.

### Treatment protocol

[^177^Lu]Lu-DOTATOC/-TATE was synthesized in-house (2/32 patients received [^177^Lu]Lu-DOTATATE). For details of the radiotracer synthesis and imaging procedures see supplement. According to the established protocol [5], patients received two treatment cycles at intervals of 8–12 weeks, with a fixed treatment activity and subsequent extension by two further cycles, if necessary. Patients received pre-therapeutic antiemesis prophylaxis with ondansetron 8 mg i.v. 30 min before the administration of [^177^Lu]Lu-DOTATOC/-TATE. An infusion of 1000 mL amino acid solution was started for renal protection and maintained until 4 h after the injection with subsequent infusion of 0.9% sodium chloride (NaCl) solution. No furosemide was administered.

### Toxicity assessment

Hematological and creatinine levels were monitored at each day of admission and after the end of PRRT every two weeks in outpatient for 8–10 weeks. To assess hemato- and nephrotoxicity, lab values during each treatment cycle and last available values were classified according to Common Terminology Criteria for Adverse Events (CTCAE) 5.0 [21]. Median time interval between initiation of treatment and last available blood values was 8.5 months (0—77).

### Dosimetry

All patients underwent imaging after treatment, which included at least one planar image of the head on the day after administration of [^177^Lu]Lu-DOTATOC/-TATE and a quantitative SPECT/CT scan after 1 to 6 days to assess and document activity uptake in tumor lesions. Additional planar images were obtained at the discretion of the treating physician.

Tomographic images were acquired using a Siemens Symbia T2 until 2018 and a Siemens Intevo Bold system thereafter. Data were acquired with medium energy collimators and a central 20% photopeak window at 208 keV between 10% scatter windows and reconstructed with scatter and attenuation correction (3D-OSEM, 6 subsets, 6 iterations and 6 mm Gaussian filter until 2018, XSpect quant, 1 subset, 48 iterations and 6 mm Gaussian filter thereafter). Planar images were acquired with a Siemens Symbia E or a Siemens Intevo Bold scanner with medium energy collimators and 20% window at 208 keV.

Radiation absorbed doses in the first treatment cycle were calculated in selected tumor lesions in 17 patients whose imaging was sufficient for dose determination. In 14 of the 17 individuals with at least two planar images on different time points, the time integrated activity was calculated using the effective half-life derived from planar imaging and the absolute activity measured by SPECT/CT. In three individuals without half-life information, doses were estimated using only SPECT/CT information according to the procedure described in [22]. Uncertainty in dose determination mainly arises from the estimation of the accumulating mass and inhomogeneity of the activity uptake that is regularly visible in PET/CT but not discernible in SPECT/CT due to lower spatial resolution. Only in a few exceptional cases did the treated meningiomas appear in CT or MR as well-demarcated homogeneous tissue with reliably measurable volume. Therefore, baseline PET was used to approximate the intratumoral distribution pattern, under the assumption that spatial uptake of the therapeutic agent during PRRT follows the same pattern as seen in diagnostic PET imaging, differing only by a constant factor.

### Statistical analysis

GraphPad Prism 10.3.1 was used for statistical analyses (GraphPad Software, San Diego, CA). Prior number of therapies and baseline PET parameters are presented as median with ranges. For PFS determination, time interval between initiation of PRRT and radiographic progression in magnetic resonance imaging was calculated. OS was defined as interval between initiation of PRRT and patient death. Uni- and multivariable cox regression analyses were performed to examine associations of different baseline parameters with PFS and OS. The Kaplan–Meier method was employed to construct survival curves, and the log-rank test was used for comparisons. Hazard ratio (HR) is presented with its 95% confidence interval (95% CI). Statistical significance was defined as *p* < 0.05.

## Results

### PET and treatment parameters

All figures given in this paragraph are median (minimum–maximum) values of the variables. The quantities measured in the baseline PET were administered activity, 113 (60–231) MBq; SUV_max_, 25 (5–297); SUV_peak_, 13 (3–153; SUV_mean_, 4 (1–57); SSTR-TV, 25 (5–363) cm^3^ and TL-SSTR, 159 (9–1884) cm^3^. The respective data in the follow up PET scan were administered activity, 109 (74–149) MBq; SUV_max_, 26 (9—106); SUV_peak_, 15 (6—47); SUV_mean_, 5 (2—22); SSTR-TV, 43 (13—379) cm^3^ and TL-SSTR, 235 (57—1197) cm^3^. Patients received 2.5 (1–8) treatment cycles with a median activity of 7.6 GBq/cycle (3.7–18.7) of [^177^Lu]Lu-DOTATOC/-TATE every 8 −12 weeks. The median cumulative activity was 22.4 (6.9–60.3) GBq. Median follow-up time was 34 months.

### Adverse events

**Fig. 3:**
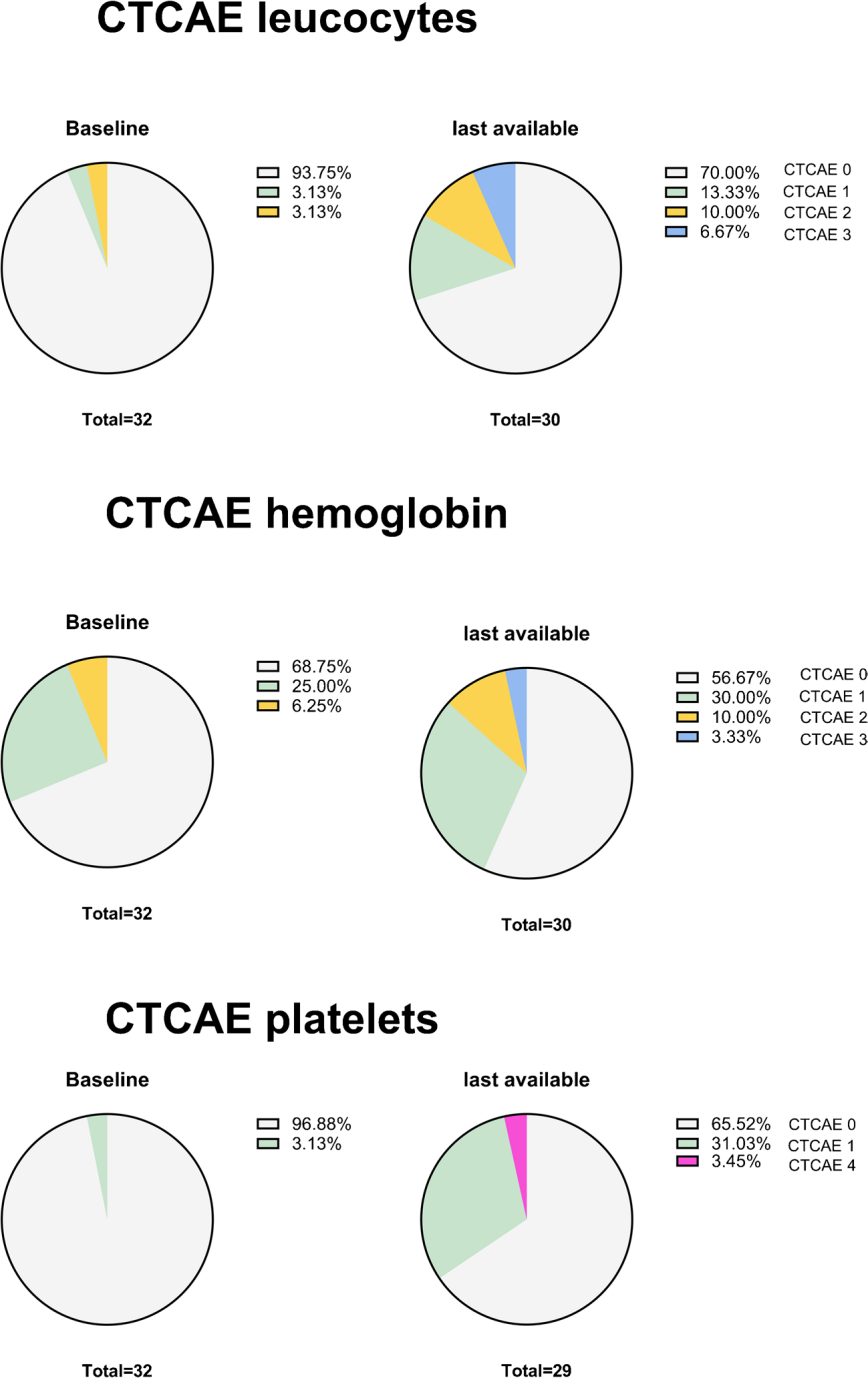
Hematotoxic adverse events at different time points during PRRT and follow-up classified according to CTCAE Version 5

Prior to PRRT, 2 patients (6%) had grade I-II leucopenia, 10 patients (31%) had grade I-II anemia, and 1 patient (3%) had grade I thrombocytopenia. No patient exhibited grade III or IV hematologic toxicities prior to therapy. During the entire monitoring period, 39 events occurred, including both newly developed toxicities and worsening of pre-existing conditions. These events were counted individually, meaning that patients who developed more than one new or progressive toxicity (e.g., both anemia and thrombocytopenia) were counted more than once. Specifically, 7 patients experienced two distinct toxicities, and 5 patients developed toxicities across all three hematologic lineages. 27/39 (69%) were of grade I (6 × leucopenia, 8 × anemia, 13 × thrombopenia), 7/39 (18%) of grade II (4 × leukopenia, 2 × anemia, 1 × thrombopenia) and 4/39 (10%) were of grade III (3 × leucopenia, 1 × anemia). Only one patient (3%) developed new grade IV thrombopenia. Of the new-onset toxicities, 14/39 (36%) (6 × leucopenia, 3 × anemia, 5 × thrombopenia) were reversible, with 13/14 (93%) returning to baseline levels. In one patient, we observed transient grade III toxicity that began to resolve by the last follow-up, but did not return to baseline level. In total, 25 non-reversible toxicities were observed during the follow-up period, 21/25 of which were grade I or II (Fig. 3 and Supplement).

**Fig. 4:**
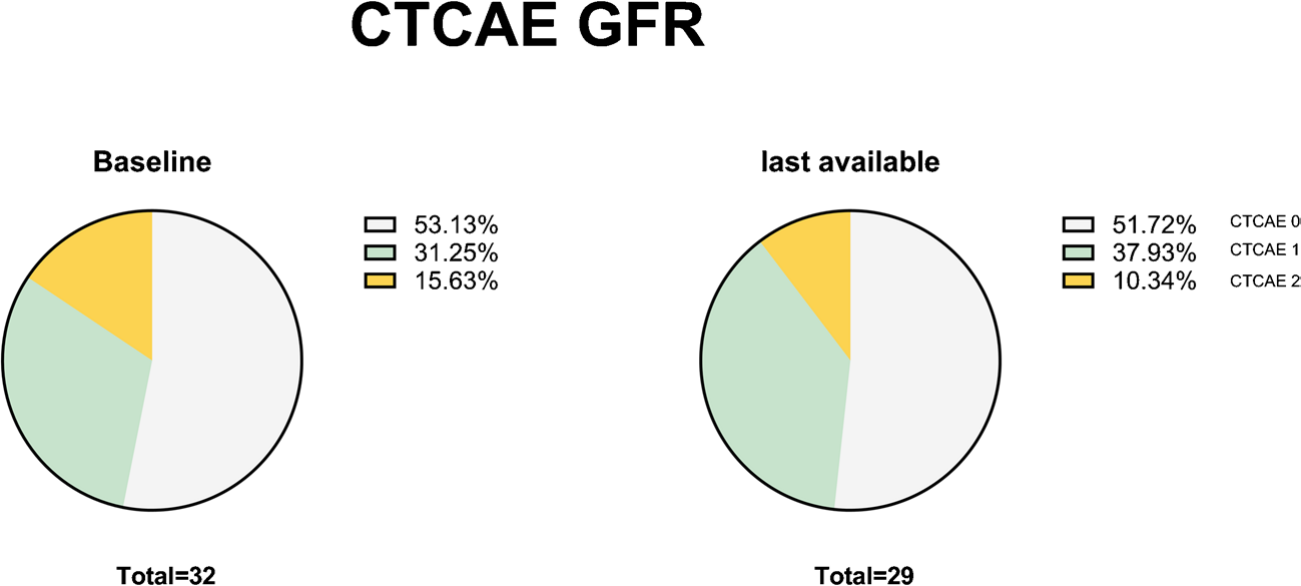
Nephrotoxic Adverse Events at different time points during PRRT and follow-up classified according to CTCAE Version 5

With regard to renal toxicity prior to treatment, we identified 10/32 (31%) patients with grade I and 5/32 (16%) with grade 2 renal dysfunction. The median pre-treatment eGFR was 92 (43—123) ml/min/1.73 m^2^. No grade III or IV renal dysfunction was observed before treatment. During PRRT, 6 patients developed new renal toxicities, all of which were grade I. 2/6 (33%) newly developed renal dysfunctions were reversible at the time of the last follow-up (Fig. 4).

### Efficacy and outcome

#### Only higher tumor grading (WHO) is associated with shorter progression-free survival

**Fig. 5:**
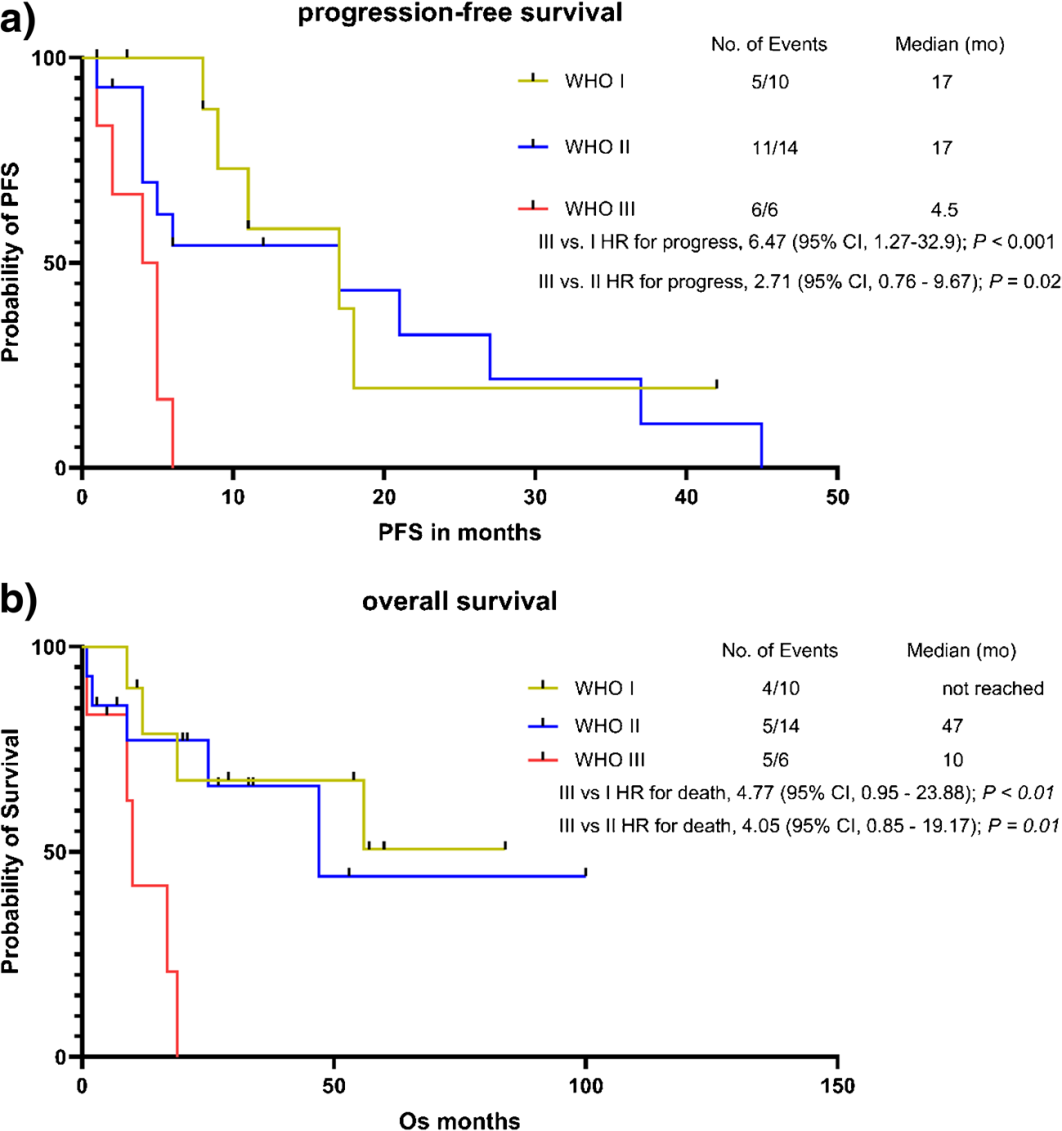
Kaplan-Meyer analysis for (**a**) progression-free survival (PFS) and (**b**) overall survival (OS) divided by WHO grading (I-III). HR Hazard ratio. CI Confidence interval. For 2/32 patients tumor grading was not available (n/a), these patients are not represented in the graphs for PFS and OS. One death occurred in the n/a group

The median PFS across all tumor grades was 9 months. Patients with WHO grade III had a significantly shorter PFS with 4.5 months, compared to WHO grade I with 17 months (HR 6.47, 95% CI 1.27–32.9, *p* < 0.001) or WHO grade II with 17 months (HR 2.71, 95% CI 0.76—9.67, *p* = 0.02). There was no significant difference in PFS for patients with WHO grade I vs. WHO grade II meningioma (HR 0.76; 95% CI 0.28—2.10, *p* = 0.6) (Fig. 5). The PFS at six months (PFS6) across all WHO grades was 58%, with a grade-specific PFS6 of 100% for WHO grade I, 54% for WHO grade II and 0% for grade III tumors. Combining grade II and III tumors, PFS6 was 37%.

Univariable cox regression analysis revealed a significantly shorter PFS for higher baseline AP levels *(per 10,* HR 1.23; 95% CI 1.02—1.46, *p* = 0.02) and higher WHO grade (HR 2.77; 95% CI 1.32—6.21; *p* = 0.01). Further multivariable analysis confirmed only higher WHO grade (HR 2.48; 95% CI 1.16—5.64; *p* = 0.02) as an independent predictor for progression. 6/32 patients were taking anticonvulsants, which could potentially lead to increased AP levels. Mann–Whitney test showed no difference between AP levels of patients taking anticonvulsants and those not taking anticonvulsants (U = 75.5, *p* = 0.9). Baseline levels of CRP, AST, bilirubin, LDH and creatinine, age at therapy and the quantitative PET parameters in baseline scan showed no significant association with PFS (Table 2).

**Table 2:** Univariable and multivariable cox regression for progression-free survival

	Univariable			Multivariable		
BL clinical variables	HR	95% CI	P	HR	95% CI	P
LDH (U/l), per 100	1.70	0.82—2.99	0.1			
AST (U/l)	0.96	0.87—1.04	0.4			
Bilirubin (mg/dl)	0.50	0.06–3.55	0.50			
CRP (mg/dl)	1.19	0.88—1.46	0.2			
*AP (U/l),* per 10	*1.23*	*1.02—1.46*	0.02*	1.18	0.99—1.40	0.05
Age	1.01	0.97—1.05	0.6			
Creatinine	0.47	0.06—3.14	0.4			
*WHO grade*	*2.77*	*1.32—6.21*	0.01*	*2.48*	*1.16—5.64*	0.02*
BL PET parameter						
SSTR-pos. TV, per 100	0.54	0.14—1.28	0.3			
TL-SSTR, per 100	0.96	0.89—1.08	0.9			
SUVmean	1.02	0.97—1.06	0.3			
SUVpeak	1.01	0.99—1.03	0.3			
SUVmax	1.01	0.99—1.01	0.2			
Δ PET parameter						
*Δ SSTR-pos. TV*	*1.02*	*1.00—1.04*	0.02*			
Δ TL-SSTR	1.01	1.00—1.03	0.06			
Δ SUVmean	1.02	0.99—1.06	0.2			
Δ SUVpeak	1.02	0.99—1.05	0.1			
Δ SUVmax	1.00	0.97—1.04	0.99			

BL baseline, Δ difference, HR hazard ratio, CI confidence interval, CRP C-reactive protein, AST aspartate aminotransferase, AP alkaline phosphatase, LDH lactate dehydrogenase, eGFR estimated glomerular filtration rate, SUV standardized uptake value, SSTR-pos. TV somatostatin receptor positive tumor volume, TL-SSTR total lesion SSTR expression. Asterisk marks significant results

#### Higher baseline LDH and CRP levels and higher WHO grade are associated with shorter overall survival

15 of 32 (47%) patients died during the follow-up period with a median survival of 49 months. Patients with WHO III meningioma showed a significantly shorter OS of 10 months compared to patients with WHO I (not reached; HR 4.77; 95% CI 0.95–23.88, *p* < 0.01) or WHO II meningioma (47 months; HR 4.05; 95% CI 0.85–19.17, *p* = 0.01). There was no significant difference in OS between patients with WHO grade I vs. WHO grade II meningioma (HR 0.75; 95% CI 0.20—2.77, *p* = 0.7) (Fig. 5). The overall survival rate at 12 month (OS12) was 72%. Univariable cox regression analysis showed a significant association with a higher risk of death for higher baseline LDH levels (*per 100*, HR 2.13; 95% CI 0.96–4.08; *p* = 0.03), higher baseline CRP (HR 1.31; 95% CI 1.01—1.64; *p* = 0.02) and a higher WHO grade at the time of therapy (HR 2.67; 95% CI 1.18—6.38; *p* = 0.02). Baseline levels of AP, AST, bilirubin, creatinine, as well as age at therapy and the quantitative PET parameters in baseline scan showed no significant association with OS (Table 3). Due to the small number of events, no multivariable analysis was conducted for OS.

**Table 3:** Univariable cox regression for clinical and PET parameters at baseline for Overall Survival (OS)

BL clinical variables	Univariable		
	HR	95% CI	P
*LDH (U/l), per 100*	*2.13*	*0.96—4.08*	0.03 *
AST (U/l)	0.97	0.86—1.06	0.5
Bilirubin (mg/dl)	0.49	0.03—6.38	0.6
*CRP (mg/dl)*	*1.31*	*1.01—1.64*	0.02 *
AP (U/l), per 10	1.13	0.90—1.38	0.2
Age (y)	1.02	0.98—1.06	0.4
Creatinine (mg/dl)	0.35	0.02–3.81	0.4
*WHO grade*	*2.67*	*1.18—6.38*	0.02 *
BL PET parameter			
SSTR-pos. TV, per 100	0.67	0.21—1.31	0.4
TL-SSTR, per 100	0.99	0.88—1.09	0.9
SUVmean	1.01	0.94—1.06	0.7
SUVpeak	1.01	0.98—1.02	0.6
SUVmax	1.00	0.99—1.01	0.7
Δ PET parameter			
Δ SSTR-pos. TV	1.02	1.00—1.04	0.05
Δ TL-SSTR	1.01	1.00—1.02	0.05
Δ SUVmean	1.03	0.99—1.07	0.1
Δ SUVpeak	1.04	1.01—1.11	0.06
Δ SUVmax	1.03	0.98—1.10	0.3

BL baseline, Δ difference, HR hazard ratio, CI confidence interval, CRP C-reactive protein, AST aspartate aminotransferase, AP alkaline phosphatase, LDH lactate dehydrogenase, eGFR estimated glomerular filtration rate, SUV standardized uptake value, SSTR-pos. TV somatostatin receptor positive tumor volume, TL-SSTR total lesion SSTR expression. Asterisk marks significant results

#### Increase of SSTR-TV is associated with shorter survival

For 13 cases, follow-up PET scans were available. The follow-up PET scans were conducted after a median of 2 cycles of PRRT (range 1–4) and 177 days (range 118–215) from the baseline scan. Univariable Cox regression revealed that only the increase of SSTR-TV showed a significant association with a shorter PFS (HR 1.02; 95% CI 1.00–1.04; *p* = 0.02), for OS the increase of SSTR-TV showed a trend towards significance (HR 1.02.; 95% CI 1.00–1.04; *p* = 0.05). The % change in SUV_max_, SUV_peak_ and TL-SSTR showed no significant association with PFS or OS (Table 2 and 3).

### Dosimetry

**Fig. 6:**
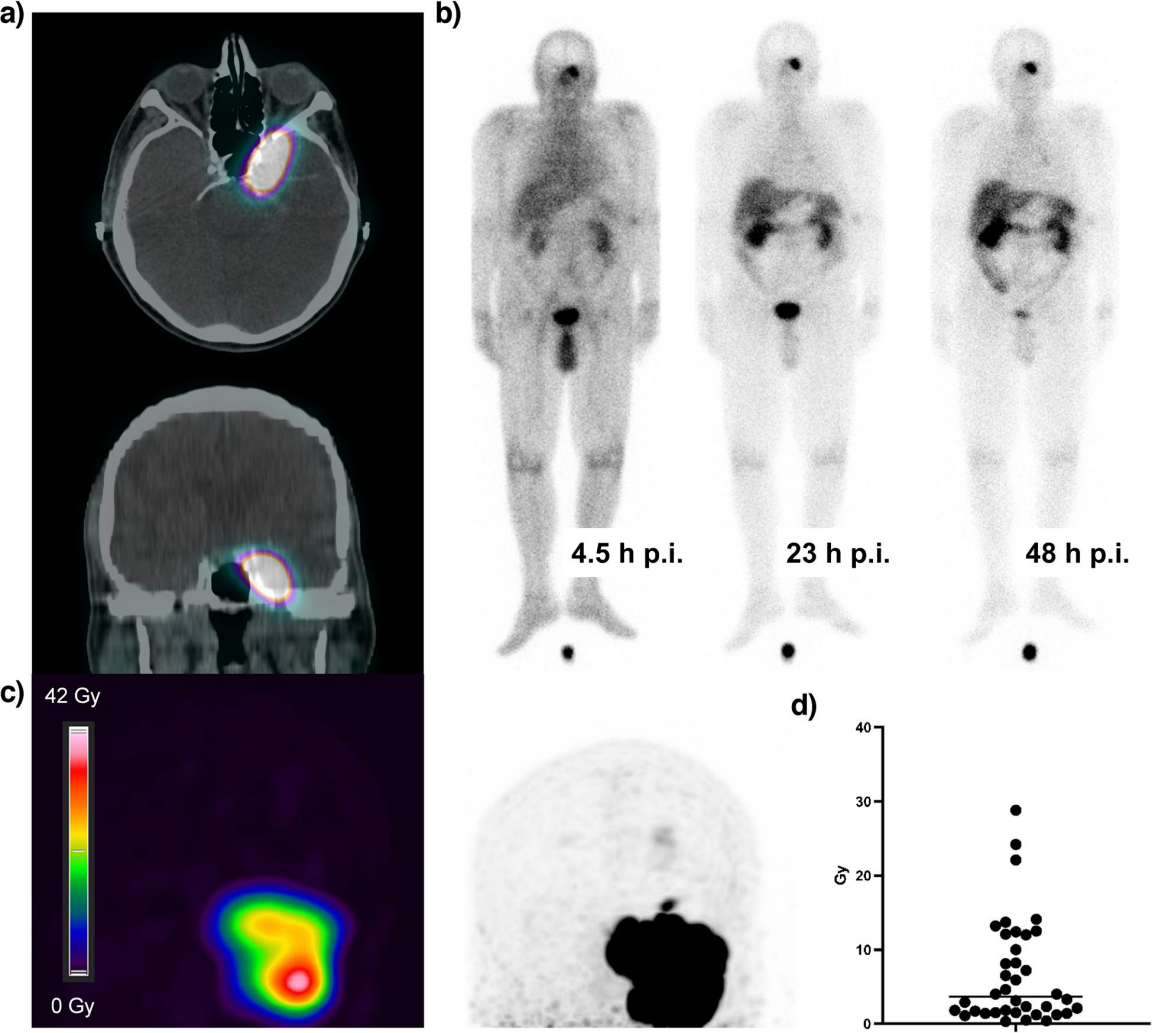
(**a**) Representative case with posttherapeutic SPECT/CT images, transversal and frontal view. (**b**) Posttherapeutic whole-body scintigraphy after 4.5 h, 23 h and 28 h post infusionem (p.i.) shows intensive radionuclide uptake in the lesion. (**c**) Illustrative presentation of intratumoral distribution of radiotracer in initial PET Scan, used to estimate uptake heterogeneity and tumor mass for dosimetric purposes. (**d**) Scattered plot of calculated lesion dose. Each dot represents the calculated mean dosage for a single lesion. Calculations were performed in 17 patients for a total of 38 lesions. Horizontal line represents median value

To estimate the radiation dose, dosimetric calculations were performed in 17 cases, covering a total of 38 lesions. The mean dose achieved in a lesion per cycle was calculated to be a median of 3.7 Gy (0.3—28.8) (Fig. 6). The lesion with the highest dose/patient (= hottest lesion) was calculated to be a median of 4.0 Gy (0.5—28.8). Cox regression analysis for the hottest lesions was performed and showed no significant association between calculated dose and PFS (HR 1.00; 95% HR 0.94—1.06, *P* = 0.9) or OS (HR 0.93; 95% CI 0.73—1.04, *P* = 0.4).

## Discussion

Meningiomas that persist after first-line surgical resection and second-line external radiotherapy pose a clinical challenge, as there are no established third-line therapies [23]. Meningiomas are known to have a high level of SSTR expression on the cell surface [7, 8]. This molecular profile enables SSTR-directed radionuclide therapy with β- emitting radionuclides, resulting in targeted irradiation of tumours cells [6]. In other SSTR-expressing tumor entities, such as neuroendocrine tumors, SSTR-guided PRRT has already been demonstrated to have good efficacy with a favourable safety profile [5]. Our data confirm the good safety profile of PRRT in patients with recurrent or progressive meningiomas and survival analysis indicates that PRRT may also have a favourable efficacy even in WHO II-III meningiomas. Regression analyses identified the WHO tumor grade as the strongest predictor of PFS and OS. Among the laboratory parameters, elevated LDH and CRP may be associated with shorter OS, while baseline PET parameters appear to have no prognostic value for OS or PFS.

The toxicities observed in our cohort were predominantly mild, with 87% of all bone marrow and renal toxicities classified as CTCAE grade I or II, 36% of which were transient in character. All new renal toxicities occurring during PRRT were of grade I. These findings are consistent with prior studies of PRRT in different tumor entities, which also reported predominantly low-grade toxicities and limited renal side effects [5, 10, 11, 24, 25]. The largest analysis of patients with recurrent meningioma who underwent PRRT to date is the meta-analysis by Mirian et al., involving 111 patients. The analysis by Mirian et al. confirms a favourable safety profile of PRRT, showing a predominance of grade I/II hepatotoxicities and only one transient case of grade IV renal toxicity [12]. Comparative data with other systemic therapies underscore the favourable toxicity profile of PRRT. It should be noted that one patient with multifocal WHO grade III meningioma died shortly after the first cycle of PRRT. However, this patient was already severly ill before initiation of treatment. Therefore, it is likely that the patient's death is not attributable to the therapeutic intervention itself, but rather to the advanced and progressive disease. Antiangiogenetic drugs, often investigated as alternative therapies, are often associated with more frequent and severe toxicities [26–30]. In a phase II trial of bevacizumab for recurrent and refractory meningiomas (*n* = 50), 42% of patients developed hypertension, 36% proteinuria, and 31% fatigue [26]. Similarly, a phase II study (*n* = 36) evaluating sunitinib reported severe adverse events, including intratumoral hemorrhages, thrombotic microangiopathy, and gastrointestinal perforations [27].

A RANO meta-analysis [23], which included 47 retrospective and prospective trials of active- and inactive agents, showed that the PFS6 for refractory or progressive meningiomas was 29% for patients with WHO grade I tumors and 26% for patients with WHO grade II/III tumors. As a result of this analysis, target thresholds of > 50% PFS6 for grade I and > 35% PFS6 for grades II/III meningioma have been recommended for future studies. In our cohort, the observed PFS6 was 58% across all WHO grades, with a grade-specific PFS6 of 100% for WHO grade I and 37% for combined WHO grade II/III tumors. These results are consistent with the meta-analysis of Mirian et al. [12] and exceed the established benchmarks, which may indicate meaningful disease control by PRRT. Systemic therapy with bevacizumab has demonstrated an even higher PFS6 of 90% for WHO grade I, 76% for grade II, and 45% for grade III [26], however, its adverse event profile may limit its applicability across diverse patient groups. Yet a direct comparison of PFS6 should be interpreted with caution due to differences in study design, patient selection, and treatment timing. The prospective nature of the study on bevacizumab involved predefined inclusion criteria, which likely resulted in a more selected, potentially healthier patient cohort. In addition, standardized imaging schedules and predefined response assessment protocols were used, which may have influenced the timing and consistency of progression detection compared to our analysis.

OS is a critical metric for evaluating therapeutic efficacy. However, its use in meningiomas is limited due to the typically slow growth rates and the concomitant necessity for protracted monitoring periods. The OS12 of 72% in our cohort aligns with previously reported findings [12]. The RANO meta-analysis reported a median OS (mOS) ranging from 6 to 33 months [23]. In our cohort, the overall mOS was 49 months, 47 months for grade II meningiomas, and 10 months for grade III meningiomas, while it was not reached for WHO grade I tumors. Although bevacizumab has been shown to have a beneficial effect on PFS, it has not yet been demonstrated to have a significant effect on OS. For Bevacizumab, a mOS of 35 months for grade I, 41 months for grade II, and 12 months for grade III meningioma has been reported [26], which is shorter than in our cohort for grade I and II meningiomas. Only grade III meningioma showed a shorter mOS in our cohort. However, the number of patients with grade III tumor included in our series was limited (*n* = 6), which might limit the statistical analysis. In addition to potential statistical limitations, the lack of demonstrated overall survival benefit with bevacizumab may also reflect underlying biological differences: While bevacizumab may temporarily stabilize disease progression through anti-angiogenic mechanisms, it does not exert a direct cytotoxic effect on tumor cells. This may explain improved short-term progression metrics (e.g., PFS6) without a corresponding impact on OS. In contrast, the targeted delivery of β- radiation to SSTR-positive tumor cells by PRRT, may potentially induce longer-lasting biological effects, including DNA damage and sustained tumor control.

In line with prevailing expectations, the factor that exerted the most substantial and significant impact on both OS and PFS was identified as the WHO grade at the time of treatment. The present analysis further explores the prognostic value of laboratory and imaging biomarkers as prognostic factors for disease progression and survival, which have rarely been investigated in this context [14, 16, 18]. Indeed, increased baseline AP levels showed a significant association with a shorter PFS in univariable regression analysis. However, this finding did not hold up in the multivariable analysis. As a ubiquitous enzyme typically linked to bone and liver metabolism [31], elevated AP levels may reflect an osteoplastic growth pattern. Interestingly, the single patient with bone metastasis did not show elevated baseline AP levels. Certain anticonvulsants are also known to elevate AP levels [32] and therefore might be a confounding factor. However, our analysis detected no difference in AP levels between patients receiving or not receiving anticonvulsant therapy.

Furthermore, our analysis highlights an association for elevated baseline LDH and CRP levels with a shorter OS. Research in other solid tumors has similarly shown that elevated LDH levels correlate with poor prognosis [33], potentially due to LDHs role in glycolysis and energy metabolism. Thereby LDH acts as a marker for aggressive tumor behavior. A previous study in patients with WHO grade II meningioma similarly described a higher preoperative LDH to be a predictor for shorter PFS [34]. CRP has been found to be a prognostic biomarker in several tumor entities [35–38] and may reflect a pro-inflammatory tumor microenvironment. However, given the retrospective nature of the study and the modest sample size, it is possible that even isolated comorbidities or medications may introduce bias into the biomarker analyses. Therefore, the prognostic role of different laboratory biomarkers remains exploratory and requires further investigation.

The observed association between an increase of SSTR-TV and shorter PFS is not entirely unexpected. Nonetheless, this finding supports the potential role of PET imaging for disease monitoring, especially to characterize dynamics of treatment response and progression and should be validated in larger cohorts with longitudinal PET assesments.

The broad variability in absorbed dose with a median of 3.7 Gy per cycle aligns with previous reports [39, 40] and may reflect underlying differences in tumor biology, perfusion, or receptor expression.

The single-center approach ensured consistency in patient selection, treatment protocols, and data collection, enhancing the reliability of the results. However, the study also has several limitations. First, the small number of patients limits statistical power and generalizability, particularly in the subgroup of WHO grade III meningiomas. In addition, the low number of events precluded multivariable analysis for OS. Second, the retrospective study design introduces potential selection bias. PRRT as a stand-alone treatment is applied as last-line treatment option, patients with poor clinical condition or rapid worsening may have been assigned to best supportive care as alternative approach. This selection process also included two patients, who were treated based on clear radiological and clinical findings in combination with multidisciplinary tumor board consensus, but without histopathological confirmation. While this reflects real-world clinical decision-making, it may introduce a risk of tumor misclassification. Third, follow-up imaging was not standardized, which may have confounded the assessment of PFS and OS due to variations in timing, protocol and reading of follow-up imaging. Lastly, data on clinical side effects and symptoms were not collected systematically, restricting the ability to evaluate PRRT´s impact beyond radiographic findings.

For future studies, the incorporation of the new WHO classification from 2021, which includes molecular markers, may help to identify patients who will benefit from PRRT even more accurately. Moreover, prospective clinical trials are essential to further evaluate the efficacy and safety of PRRT in this patient population. Currently, one such prospective study has been initiated (LUMEN-1 study, NCT 06326190), marking an important step toward generating higher-level evidence for this therapeutic approach.

## Conclusion

PRRT is a well-tolerated treatment option and appears to be effective in patients with recurrent meningiomas, with both PFS and OS exceeding threshold values recommended by the RANO group for this patient population. WHO tumor grade is the strongest predictor of PFS and OS, while baseline PET parameters do not appear to provide prognostic value. Further studies on dose-dependent efficacy, different histological classifications and treatment regimens are needed to achieve a more effective and individualised approach.

## Supplementary Information

Below is the link to the electronic supplementary material.

Supplementary file1 (DOCX 201 KB)

## Data Availability

The main data presented in this study are available in the article. Detailed information about the image analysis or the overall survivals of the subjects presented in this study are available on reasonable request from the corresponding author.
