# Supplementary material for: SSTR-directed peptide receptor radionuclide therapy for recurrent meningiomas: analysis of safety, efficacy and prognostic factors
Source: Eur J Nucl Med Mol Imaging. 2025 Jun 2;53(1):116–27. doi: 10.1007/s00259-025-07336-6 (PMC12660467; doi:10.1007/s00259-025-07336-6)

# **7. Supplement**

## **Radiotracer synthesis for SSTR PET**

The [^68^Ga]Ga-DOTATOC tracer for SSTR PET/CT was prepared in the dept. of radiopharmaceutical chemistry, University Hospital Würzburg, shortly before the examination. The production was carried out according to GMP (Good Manufacturing Practice) guidelines. A GRP® module (SCINTOMICS GmbH, Fürstenfeldbruck, Germany) was used, connected to a gallium-68 generator (Eckert und Ziegler, Berlin, Germany) and equipped with a disposable cassette kit (ABX, Radeberg, Germany). An eluate of [^68^Ga]GaCl3 ([^68^Ga]Ga chloride) in 0.1 M hydrochloric acid (HCl) was transferred to a cation exchange cassette, diluted with 5 N NaCl and added to a solution of 20 µg DOTATOC (ABX, Radeberg, Germany) in HEPES buffer. After heating at 125°C for 6 minutes, the liquid was transferred to a SepPak C18 cartridge, washed with water and eluted with 50% ethanol. After sterile filtration (0.22 µm), the eluate was diluted with phosphate buffer to a total volume of 15 ml. Quality control included verification of radiochemical purity using high performance liquid chromatography and thin layer chromatography. In addition, the pH was determined, an endotoxin test and a bubble point test were performed.

## **Examination procedure SSTR PET/CT**

PET scans were acquired with a Siemens Biograph mCT 64 scanner or a Siemens Biograph mCT 128 Flow Edge R (Siemens, Knoxville, USA) in a three-dimensional mode. The examination procedure was based on the recommendations of the German Society of Nuclear Medicine (DGN) and EANM [41, 42]. Image acquisition took place after 45 – 60 min after radiotracer injection. The iterative reconstruction of the images was performed using an Ordered Subset Expectation Maximalisation (OSEM) algorithm with 3 iterations and 21 (Biograph mCT 128 Flow) or 24 (Biograph mCT 64) subsets. This was followed by post-reconstructive smoothing with a Gaussian filter (2 mm Full Width at Half Maximum (FHWM); Siemens TrueX (+ TOF ultra HD in mCT 128). The reconstructed PET images have a spatial resolution of 4.7 mm FWHM (Biograph mCT 128 Flow) to 5.7 mm FWHM (Biograph mCT 64).

## **7.3 Patients comorbidities and comedications**

| Pat. no. | relevant comorbidities | | medications | |  |
| --- | --- | --- | --- | --- | --- |
| 1 | reflux | oxcarbazepin, hydrocortisone, omeprazol | | | |
| 2 | sleep apnoea, hypothyroidism, depression | pregabaline, venlafaxine, lamotrigine, levothyroxine, doxepin | | | |
| 3 | dementia, acute urinary tract infection | duloxetine, valproat, zolpidem, quetiapine, metamizole, dexamethasone, tilidine, nitrofurantoin, levetiracetam | | | |
| 4 | arterial hypertension, coronary heart disease | acetylsalicylic acid, molsidomine, metoprolol, amlodipine, atorvastatin, temazepam | | | |
| 5 | arterial hypertension, coronary heart disease, glaucoma, dislipoproteinaemia | bisoprolol, ramipril, amlodipine, simvastatin, acetylsalicylic acid, levetiracetam, oxcarbazepin, dorzolamide, lacosamide, torasemide, pantoprazole | | | |
| 6 |  | pregabaline, lacosamide, ibuprofen | | | |
| 7 | immunogenic thrombocytopenia arterial hypertension | benazepril | |  |  |
| 8 | arterial hypertension, history of malign melanoma and breast cancer | ramipril, pantoprazole, lamotrigine, levetiracetam, simvastatin, diclofenac, cortisone | | | |
| 9 | arterial hypertension, diabetes mellitus, depression | eplerenone, metoprolol, ramipril, metformin, glimepiride, pantoprazol, methylprednisolone, levetiracetam, lacosamide, reboxetine | | | |
| 10 | dislipoproteinaemia, arterial hypertension | pantoprazole, levetiracetam, amlodipine, simvastatin, enalapril, fraxiparine | | | |
| 11 | arterial hypertension, history of thyroidectomy | levothyroxine, bisoprolol, valsartan | | | |
| 12 | history of osteosarcoma, Woff-Parkinson-White syndrome, bicuspid aortic valve | levetiracetam, valproat, pantoprazol, lacosamide, everolimus | | | |
| 13 | Hypothyroidism, adjustment disorder, neurogenic bladder disorder, Incomplete paraplegic syndrome | levothyroxin, dexamethasone, pregabaline, mirtazapine, ondansetron, tamsulosin, citalopram, dimenhydrinate | | | |
| 14 |  | no | |  |  |
| 15 | arterial hypertension, history of stroke, hemiplegia | mirtazapine, pantoprazole, simvastatin, ramipril, clopidogrel, metamizole, prothipendyl, citalopram | | | |
| 16 | Arterial hypertension, hyperlipoproteinemia | amlodipine, atorvastatin, lisinopril, piretanide, bisoprolol, pregabaline | | | |
| 17 | history of vestibular schwannoma, history of ALL, arterial hypertension, hyperlipoproteinaemia | valproat, levetiracetam, venlafaxine | | | |
| 18 | Gorlin-Goltz syndrome, history of basal cell carcinoma and medulloblastoma | levetiracetam | | |  |
| 19 | History of colon cancer and transient ischemic attack, Parkinson’s disease | madopar, carbidopa/levodopa, levothyroxine , acetylsalicylic acid, pantoprazole, folsan, vitamin D, valproate, entacapone | | | |
| 20 | arterial hypertension, AV block III with pacemaker implantation, hypothyroidism, Incomplete paraplegic syndrome, history of transitory ischemic attack | lasoamide, levetiracetam, baclofen, acetylsalicylic acid, amlodipine, metoprolol, simvastatine, ramipril, levothyroxine | | | |
| 21 | arterial hypertension | hygroton, ramipril, levetiracetam | | | |
| 22 | diabetes mellitus, arterial hypertension, sleep apnea | empagliflozine, valsartan | | |  |
| 23 | hypothyroidism, arterial hypertension, benign positional vertigo, history of trauma-induced degeneration in premotor cortex after skull fracture | levothyroxine, pantoprazole, levetiracetam, hydromorphone, metamizole | | | |
| 24 | arterial hypertension, history of stroke | acetylsalicylic acid, simvastatin, salbutamol, pantoprazole, ramipril, amlodipine | | | |
| 25 | arterial hypertension, cardiac arrhythmia, history of prostate cancer | torasemide, nebivolol, diclofenac | | | |
| 26 | arterial hypertension, migraine, hypothyroidism | ramipril, levothyroxine | | |  |
| 27 | arterial hypertension | valsartan, metoprolol | | |  |
| 28 | benign prostatic hyperplasia | levetiracetam, tamsulosine | | | |
| 29 | arterial hypertension, hypothyroidism, depression, gastritis | lorazepam, omperazol, levothyroxine, candesartane, sertraline, mirtazapine | | | |
| 30 | depression, chronic heart disease, asthmatic disease, arterial hypertension, hypothyroidism, diabetes mellitus | milnacipran, furosemide, levothyroxine, metformin, levotiracetam, nebivolol, acetylsalicylic acid, pantoprazole, insulin, vitamin D | | | |
| 31 | arterial hypertension, history of basalioma | bisoprolol, lercanidipine, temazepam, perindopril, indapamide | | | |
| 32 | partial anterior pituitary gland insufficiency, schizophrenia, hyperlipoproteinemia, diabetes mellitus | levothyroxine, sitagliptin, metformin, levetiracetam, clozapine, hydrocortisone, simvastatin | | | |


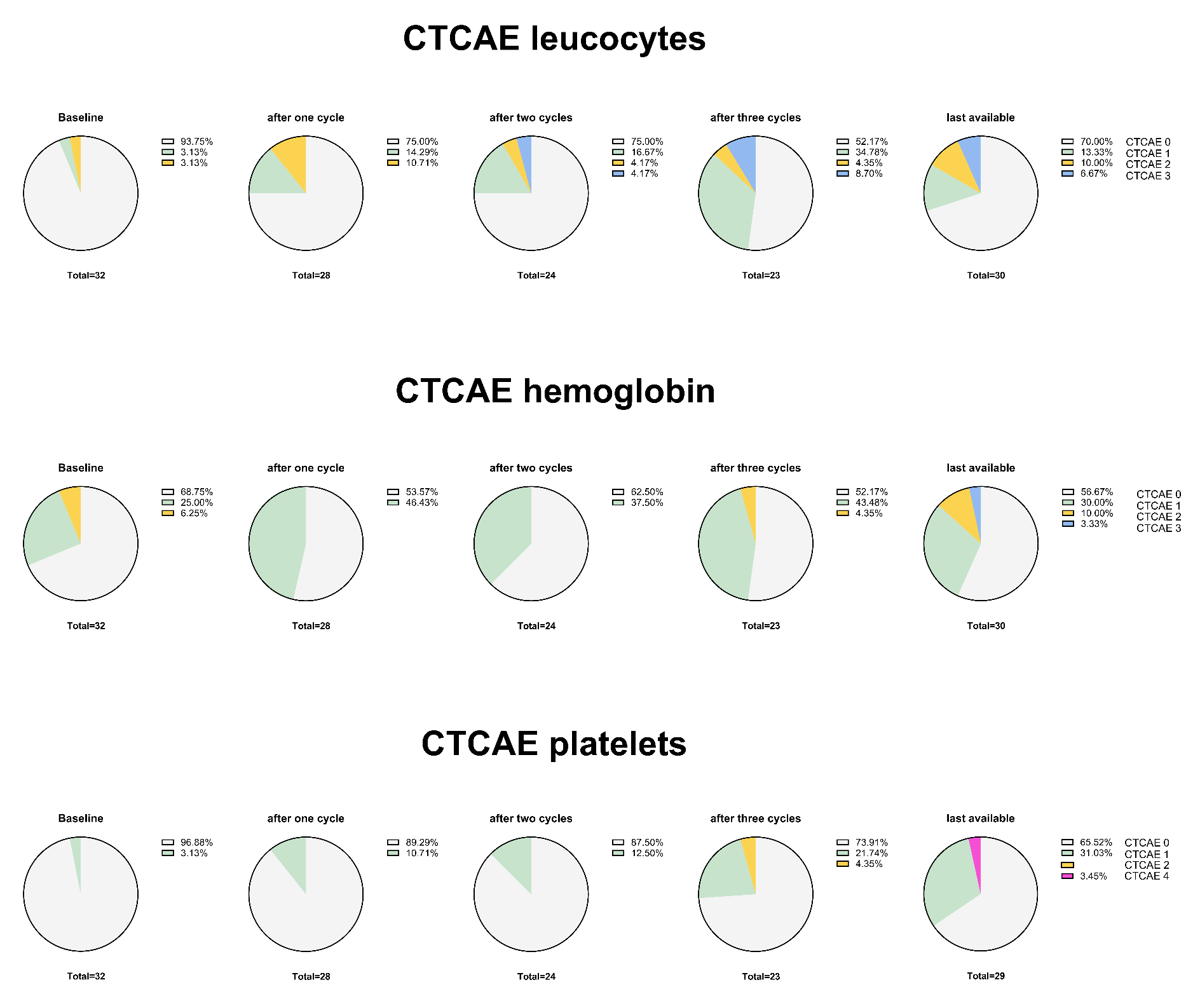

Supplement: Supplementary file 1 — Supplementary file1 (DOCX 201 KB) [file 259_2025_7336_MOESM1_ESM.docx]
